# SD Bioline malaria antigen *Pf* (HRP-2/*p*LHD) for assessing efficacy of artemisinin combination therapy against Plasmodium falciparum in pediatric patients in the Democratic Republic of the Congo

**DOI:** 10.11604/pamj.2015.22.304.6348

**Published:** 2015-11-25

**Authors:** Albert Lukuka Kilauzi, Jose Gaby Tshikuka Mulumba, Mgaywa Gilbert Mjungu Damas Magafu, Reginald Matchaba-Hove, Roy Tapera, Naoko Shimizu Magafu, Jean Jacques Muyembe Tamfum

**Affiliations:** 1 National Biomedical Research Institute (INRB), Democracy Avenue, P. O. Box 1197, Gombe, Kinshasa, the Democratic Republic of the Congo; 2 Department of Public Health Medicine, Faculty of Medicine, University of Botswana, Private bag 00713, Gaborone, Botswana; 3 School of Public Health, Faculty of Health Sciences, University of Botswana, Private Bag 0022, Gaborone, Botswana; 4 Japan Overseas Christian Medical Co-operative Services, C/o Roman Catholic Archdiocese of Tabora, Private Bag, Tabora, Tanzania

**Keywords:** Malaria, AgPf(HRP-2/pLDH) RDT, artemisinin combination therapy, Democratic Republic of the Congo

## Abstract

**Introduction:**

The emergence of *Plasmodium falciparum* resistance to artemisinin combination therapy (ACT) is a worrying development. It calls for close surveillance to monitor the efficacy of the drugs. The objectives of this study were to determine the performance of SD Bioline malaria Ag*Pf*(HRP-2/*p*LDH) 3 band Rapid Diagnostic Test (RDT) against Giemsa-stained blood smear and evaluate the suitability of this test in assessing the therapeutic efficacy of ACT in pediatric malaria patients in the Democratic Republic of the Congo (DRC).

**Methods:**

Five hundred and one patients with malaria symptoms were screened for *P. falciparum* in Kinshasa, DRC. Of the 166 patients who tested positive for *P. falciparum* at recruitment (day 0), 103 consented to participate in this study and were followed up and retested for *P. falciparum* on day 3, day 7, day 14, day 21 and day 28.

**Results:**

Sensitivity and specificity of the test were significantly high on day 0 and so were their positive and negative predictive values. Higher proportions of false positive cases were observed on the HRP-2 band irrespective of patient parasite densities during the follow up but these were barely seen on the *p*LDH band. Some patients turned positive during follow up but *p*LDH readings remained consistent with blood smear readings.

**Conclusion:**

SD Bioline malaria Ag*Pf*(HRP-2/pLDH) RDT demonstrated high performance in DRC. Thus, the test can be employed to assess the efficacy of ACT in pediatric malaria patients and prioritize areas that require the deployment of advanced testing like polymerase chain reaction (PCR).

## Introduction

Commercially available rapid diagnostic tests (RDTs) are numerous in the market. They all fall into three categories: (i) RDTs that detect the *Plasmodium falciparum* antigen histidine-rich protein 2 (HRP-2), (ii) RDTs that detect lactate dehydrogenase (*p*LDH) from *P. falciparum* and (iii) RDTs that detect *P. vivax* aldolase in human whole blood [1].

Generally, it is known that *P. falciparum (Pf)* HRP-2 remains in the bloodstream for an extended time following a successful clearance of the parasite, thus, contributing to false positive results and limiting specificity of the test category that targets *Pf*HRP-2 [2]. In contrast, *p*LDH is known to be rapidly cleared from the bloodstream and becomes undetectable at about the same time as blood smears become negative after a successful anti-malarial therapy [3, 4].

In order to monitor the success of an anti-malarial drug therapy, particularly in the presence of fever, *p*LDH-based tests seem to be more useful since they become negative soon after parasites are cleared from the blood [3]. Thus, *p*LDH-based tests better discriminate false positive results from treatment failure than HRP-2 tests. SD Bioline malaria Ag*Pf*(HRP-2/*p*LDH) RDT has been reportedly proven to be effective in reducing the rate of false positives caused by the persistence of HRP-2 after a successful drug therapy and guiding accurate prescription and monitoring efficacy of drug therapy in countries like Burkina Faso [4]. However, literature on the performance of the RDT in Kinshasa, DRC is scanty.

The objective of this study was to determine the performance of SD Bioline malaria Ag*Pf*(HRP-2/*p*LDH) 3 band RDT in symptomatic pediatric malaria patients against microscopy of Giemsa-stained blood films and to evaluate the effectiveness of SD Bioline malaria Ag*Pf*(HRP-2/*p*LDH) in monitoring the efficacy of ACT (artesunate amodiaquine) among pediatric patients in Kinshasa, DRC where *P. falciparum* transmission is characterized as high [5].

## Methods

### Study area, population and procedures

This study was conducted at Tembo Health Center in Kinshasa in DRC from October to December 2012. The health facility was chosen because of its relatively high number of pediatric patients in the 3 months preceding this study. This high number of pediatric patients was used as indicative of the availability of sufficient study participants. All pediatric patients who presented with symptoms of malaria during the study were invited to participate. After obtaining consent from patients’ parents or guardians, demographic and clinical data were collected including previous anti-malarial drug intake. Blood samples (finger prick or venous blood) for thick and thin blood films and for the SD Bioline malaria Ag*Pf*(HRP-2/*p*LDH) RDT were collected from the patients on the day of recruitment (day 0). Blood slides were stained with Giemsa 3% for 30 minutes. The first slide readings were performed at the study site while the second readings were conducted at the National Biomedical Research Institute in Kinshasa. Those with discrepant results were read by a third reader under the supervision of the principal investigator, a parasitologist, until when the final agreement was reached. Technicians reading blood slides were blinded to the results of the SD Bioline malaria Ag*Pf*(HRP-2/*p*LDH) RDT. Likewise, those reading the RDT were blinded to the results of the blood slides.

SD Bioline malaria Ag*Pf*(HRP-2/*p*LDH) RDT was performed according to the manufacturer's instructions and read after 15 minutes by the first laboratory technician and then after 30 minutes by the second laboratory technician who was blinded to readings from the first technician. In case of a discrepancy between the readings, the test was repeated. Results were recorded as follows: (i) when the 3 bands of the test kit were all colored, the test was positive for both *Pf*HRP-2 and *Pfp*LDH, (ii) when bands C and T1 were colored, the results were positive for *Pf* HRP-2 and not for *Pfp*LDH, (iii) when bands C and T2 were colored, the results were positive for *Pfp*LDH and not for *Pf*HRP-2 and (vi) when only band C was colored, the results were negative. Parasite density was computed by counting the number of parasites/200 white blood cells (WBC) and assuming a WBC count of 8,000/µl [6].

Positive cases of malaria were managed as recommended by the DRC National Malaria Control Program and World Health Organisation (WHO) [7]. To monitor the persistence of HRP-2/*p*LDH in the blood, respondents were followed for 28 days from day 0. Thus, besides day 0, blood samples for thick and thin blood films as well as for the SD Bioline malaria *Pf* (HRP-2/*p*LHD) RDT were collected on day 3, day 7, day 14, day 21 and day 28. The tests were performed following the same procedure as described above.

### Operational case definitions

Sensitivity of the RDT assay is defined herein as its ability to correctly detect blood specimens containing malaria antigen. It is the percentage of true positive (TP) malaria specimens identified by the RDT as positive divided by the number of specimens identified by the gold standard assay (Giemsa-stained blood smear) as positive (TP)+false negative (FN).

Specificity of the RDT assay is its ability to correctly discriminate specimens that do not contain malaria antigen. It is the percentage of true negative (TN) specimens identified by the RDT as negative divided by the number of specimens identified by the gold standard assay as negative (TN)+false positive (FP).

Positive predictive value (PV+) is the probability that when the test is reactive, the specimen actually contains malaria antigen. PV+ is calculated as TP/(TP+FP). Negative predictive value (PV-) is the probability that when a test is negative, a specimen does not have malaria antigen. PV- is calculated as TN/(FN+TN).

### Data analysis

Data were entered into MS Excel (Redmond, WA, USA) independently by two people. The two datasets were then compared and discrepancies were corrected to create the final version that was analyzed using IBM SPSS Statistics version 20 (Chicago, IL, USA). The performance of SD Bioline malaria Ag*Pf*(HRP-2/*p*LDH) RDT was assessed by measuring its sensitivity, specificity, positive and negative predictive values against blood smear microscopy as the gold standard [8].

The proportion of false positives among the children were estimated as a function of *Pf*HRP-2/*p*LDH antigen present in <1,000 parasites/µl, 1001 - 5,000 parasites/µl, 5,001 - 50,000 parasites/µl and in > 50,000 parasites/µl on day 3, 7, 14, 21 and 28. In other words, this represented the percentage of participants who tested positive for Ag*Pf*(HRP-2/*p*LDH) RDT while having a negative blood smear test for a given parasite density level and day of follow up. We compared groups using Fisher's exact test. The level of significance was set up at p<0.05.

### Ethical approval

This study was first approved by the DRC National Biomedical Research Institute's Research Committee. Written informed consent was sought from parents or guardians of the children who participated in this study. The parents or guardians were assured that the data would be kept confidential and used only for research purposes. Participants who tested positive for malaria were given standard malaria management as stipulated by the DRC National Malaria Control Program and WHO.

## Results

### Study participants

In total, 501 children were screened for *P. falciparum* in this study. Their mean (standard deviation) age was 26.9 (5.8) months. The youngest was 3.4 months old while the oldest was 53.2 months old. Two hundred and thirty six (47.1%) participants who were screened were females and 265 (52.9%) were males. Three hundred and thirty five (66.9%) participants tested negative for malaria while 166 (33.1%) tested positive for malaria infection. Parents/guardians of 103 children (out of the 166) gave consent to participate in the follow up part of the study, giving a response rate of 62.0%.

### SD Bioline malaria AgPf(HRP-2/pLDH) performance

The frequency distribution of the study participants by parasite density level is presented in Table 1 whereby the parasitaemia bracket 5,001-50,000/µl had the highest percentage of children i.e. 12.8%. Results of the SD Bioline malaria Ag*Pf*(HRP-2/*p*LDH) RDT performance against blood smear microscopy as the gold standard are presented in Table 2. SD Bioline malaria Ag*Pf*(HRP-2/*p*LDH) RDT exhibited high sensitivity, specificity, positive and negative predictive values. Table 3 presents the sensitivity of SD Bioline malaria Ag*Pf*(HRP-2/*p*LDH) RDT by level of P. falciparum parasitaemia. The sensitivity of SD Bioline malaria Ag*Pf*(HRP-2/*p*LDH) ranged from 85.1% to 100%. The RDT exhibited its lowest sensitivity when the parasitaemia was less than 10,000/µl.

**Table 1:** Frequency distribution of the study participants by parasite density level

Parasite density parasites/ml	Participants	Percentage (%)
0	335	66.9
≤ 1,000	47	9.4
1,001 – 5,000	10	2.0
5,001 – 50,00	64	12.8
> 50, 000	45	9.0
Total	501	100.1

The total% exceeds 100 because of rounding off

**Table 2:** SD Bioline malaria Ag*Pf* (HRP-2/*p*LDH) performance against blood smear microscopy as the gold standard

		Smear microscopy			Performance (%)			
	Results	+	-	Total	Sensitivity	Specificity	PV^+^	PV^-^
RDT Band (HRP-2/pLDH)	+	158	12	170	95.2	96.4	92.9	97.6
	-	8	323	331				
	Total	166	335	501				

RDT = rapid diagnostic test,+= positive, - = negative, PV+ = positive predictive value; PV- = negative predictive value

**Table 3:** Sensitivity of SD Bioline malaria Ag*Pf*(HRP-2/*p*LDH) by level of *P. falciparum* parasitaemia

Parasite density/ml	Microscopy	HRP-2		*p*LDH	
	+	+	Sensitivity	+	Sensitivity
0	0	12	NA	12	NA
≤ 1,000	47	40	85.1	40	85.1
1,001-5,000	10	10	100	10	100
5,001-50,000	64	64	100	64	100
> 50,000	45	44	98.0	44	98.0

NA = Not applicable

### SD Bioline malaria AgPf(HRP-2/pLDH) performance during follow up days

One hundred and three patients who were positive for *P. falciparum* on day 0 and their parents or guardians consented to participate in this study were followed up till day 28. They were 48 females and 55 males. Their mean age (standard deviation) was 31.4 (7.4) months. The youngest was 6 months old and the oldest was 50 months. After treatment, on day 3 and day 7, all patients tested negative by blood smear microscopy. On day 14, two patients became positive by blood smear microscopy and two were lost to follow up. On day 21, four more patients became positive and six were lost to follow up. On day 28, eleven other patients became positive making a total of 17 patients who became positive. These cases represented either treatment failure or new infections and six more were lost to follow up making a total of 14 children lost to follow up. The performance of SD Bioline malaria Ag*Pf*(HRP-2/*p*LDH) against blood smear microscopy by day of follow up is presented inTable 4.

**Table 4:** SD Bioline malaria Ag*Pf*(HRP-2/*p*LDH) performance by day of follow up

Day	Microscopy		HRP-2				*p*LDH			
	+	-	+	-	Sens (%)	Spec (%)	+	-	Sens (%)	Spec (%)
Day 3	0	103	103	0	NA	NA	8	95	NA	92.2
Day 7	0	103	103	0	NA	NA	2	101	NA	98.5
Day 14	2	98	95	5	100	5.1	1	99	50	100
Day 21	4	90	81	13	100	14.4	0	94	NA	100
Day 28	11	77	74	14	100	18.2	9	79	81.8	100

Sens = sensitivity, spec = specificity, NA = not applicable,+= positive and - = negative

### Proportions/percentages of false positives by parasite density and by day of follow up

**Figure 1:**
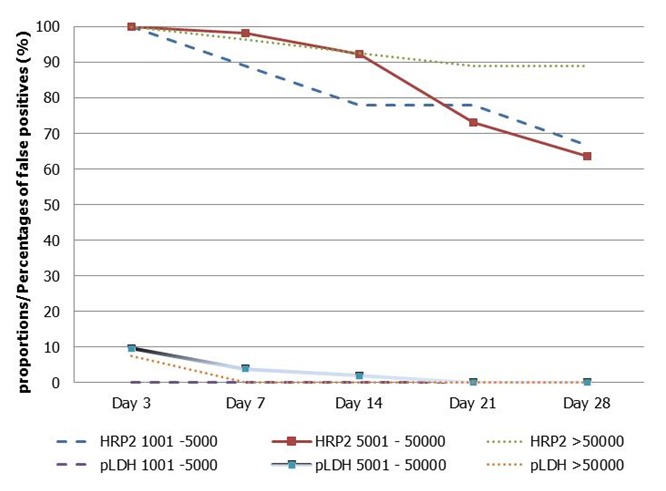
Sensitivity of HRP-2 or *p*LDH band of the SD Bioline malaria Ag*Pf*(HRP-2/*p*LDH) by parasite density in follow up days

Figure 1 compares the proportions of false positives among participants in bands HRP-2 and *p*LDH on day 3, 7, 14, 21 and 28. Band HRP-2 had higher proportions of false positives than *p*LDH. Though the proportion of false positives dropped with days of follow up until day 28 it remained significantly higher in band HRP-2. The proportions of false positives in band pLDH were significantly (*p*<0.05) lower than those in band HRP-2. In the *p*LDH band, except for patients having parasite densities of 5,001-50,000/µl where a few cases of false positives were observed till day 14, no single case of false positives was observed among patients with parasite densities ≤ 1,000/µl on day 3, 7, 14, 21 and 28. There were no false positives among children with parasite density ≤ 50,000/µl on 7, 14, 21 and 28 in band *p*LDH.

## Discussion

The main finding of this study is that the performance of SD Bioline malaria Ag*Pf*(HRP-2/*p*LDH) RDT among pediatric patients with symptomatic malaria in Kinshasa, DRC was high. The test attributes for testing and monitoring the efficacy of ACT treatment among pediatric patients in the population studied were reasonably high. The sensitivity and specificity of the test were found to be high. The corresponding positive and negative predictive values were also high indicating the high performance of this test.

Although the sensitivity of the test went a bit down to 85.1% among patients with parasite densities ≤ 1,000/µl, this study showed sensitivity of about 100% for parasite densities > 1,000/µl. Similar results are found in the literature [9, 10]. These results indicate that when using SD Bioline malaria Ag*Pf*(HRP-2/*p*LDH) RDT in this population, the probability of testing positive when one truly has malaria is more-or-less 100% which indicates high performance of this test in Kinshasa, DRC.

The evaluation of the performance of SD Bioline malaria Ag*Pf*(HRP-2/*p*LDH) for monitoring the efficacy of ACT treatment showed higher proportions of false positives with respect to the HRP-2 band irrespective of patient parasites density and day of follow up. This means that readings from the test band HRP-2 are not suitable for monitoring the efficacy of ACT treatment. The fact that false positive cases were barely seen on the *p*LDH band makes this band the most suitable for monitoring the efficacy of ACT treatment. Though few cases of false positives were observed among patients with parasite densities of 5001- 50,000/µl up to day 14, no single case of false positive was observed among patients with other parasite densities up to the end of the follow up. The concomitant capability of the *p*LDH band to turn negative when blood smear microscopy is negative or positive when blood smear microscopy is positive as demonstrated herein is an attribute that makes SD Bioline malaria Ag*Pf*(HRP-2/*p*LDH) RDT a suitable test for monitoring the efficacy of ACT treatment in this area of high*P. falciparum* transmission.

Considering the high level of *P. falciparum* transmission in Kinshasa, the major limitation of this test is its inability to differentiate between true treatment failure and reinfection cases as in total 17 cases (16.5%) became positive before the end of the study after initial successful treatment. Whether these were true cases of treatment failure or cases of new infection, only a polymerase chain reaction (PCR) procedure can differentiate. PCR is not easily accessible in this resource-poor country. Thus, SD Bioline malaria Ag*Pf*(HRP-2/*p*LDH) RDT can serve as a guide and a prioritization tool for monitoring the efficacy of ACT treatment in communities were the study was conducted. Once decreased ACT efficacy is suspected in a community, PCR should then be employed to discriminate between treatment failure and reinfection.

## Conclusion

SD Bioline malaria Ag*Pf*(HRP-2/*p*LDH) RDT has been shown to have high sensitivity, specificity, positive and negative predictive values among pediatric patients with symptomatic malaria in Kinshasa, DRC. The RDT's relatively good attributes make it suitable for use as a guide in the monitoring of the efficacy of ACT treatment among pediatric patients in resource-poor areas like Kinshasa, DRC.
